# Unraveling Network Pharmacology‐Based Therapeutics of Anthranilate Sulfonamides via Sirtuins/FOXO3a Cascade in Alzheimer's Disease

**DOI:** 10.1111/jnc.70377

**Published:** 2026-02-19

**Authors:** Waralee Ruankham, Veda Prachayasittikul, Ratchanok Pingaew, Wilasinee Jeungprasopsuk, Tanawut Tantimongcolwat, Virapong Prachayasittikul, Supaluk Prachayasittikul, Kamonrat Phopin

**Affiliations:** ^1^ Department of Clinical Chemistry, Faculty of Medical Technology Mahidol University Bangkok Thailand; ^2^ Center for Research Innovation and Biomedical Informatics, Faculty of Medical Technology Mahidol University Bangkok Thailand; ^3^ Department of Chemistry, Faculty of Science Srinakharinwirot University Bangkok Thailand; ^4^ Department of Clinical Microbiology and Applied Technology, Faculty of Medical Technology Mahidol University Bangkok Thailand

**Keywords:** Alzheimer's disease, anthranilic acid, network pharmacology, neuroprotection, sirtuins/FOXO3a cascade, sulfonamide

## Abstract

Sulfonamide‐based compounds have been a clinically attractive scaffold for drug development and proven as antioxidant and antimicrobial agents, but their pharmacological derivatives containing anthranilates (SA**1–4**) and therapeutic targets are not clearly clarified. To unravel the neuroprotective roles and underlying mechanisms of SA**1–4** against oxidative injury and healthy longevity crosstalk, a combination of in vitro experiments, in silico modeling, and network pharmacology was employed. Pretreatment with SA**1–4** in human neuronal SH‐SY5Y cells significantly regulated sirtuins (SIRTs)/forkhead box class O 3a (FOXO3a)‐mediated longevity signaling pathway via targeting endogenous antioxidant enzymes (i.e., superoxide dismutase 2 [SOD2] and catalase [CAT]), apoptotic cascades (i.e., Bcl‐2‐associated X‐protein [BAX] and B‐cell lymphoma‐2 [BCL‐2]), mitochondrial balance, and ultimately led to the neuronal rescue. Molecular docking simulations support the possibility of the SA**1–4** modulatory effect within the active binding site of SIRT1. Importantly, in silico predictions of pharmacokinetic profiles suggested that the synthetic compounds possessed preferable drug‐like properties, good oral bioavailability, and safety profiles. Network pharmacology also revealed the involvement of SA**1–4** and key targets‐regulated SIRTs in neurodegeneration, including non‐amyloidogenic cascade, tau phosphorylation, calcium homeostasis, insulin‐mediated glucose uptake, and neuroinflammation. Therefore, SA**1–4** exert promising multi‐target therapeutic strategies against oxidative damage, potentially offering alternative anti‐Alzheimer candidates for further clinical neurodegenerative and anti‐aging therapeutics.

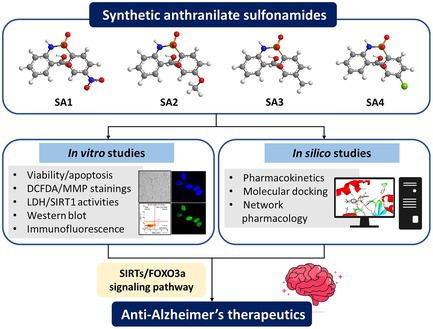

## Graphical abstract

Anthranilates sulfonamides (SA**1–4**) showed neuroprotective activity against human neuronal SH‐SY5Y cells under oxidative stress. The data represented significantly regulated sirtuins (SIRTs)/forkhead box class O 3a (FOXO3a)‐mediated longevity signaling pathway via targeting endogenous antioxidant enzymes, including superoxide dismutase 2 (SOD2) and catalase (CAT), apoptotic cascades (BAX and BCL‐2), and mitochondrial balance. Additionally, in silico investigation of pharmacokinetic profiles indicated that the synthetic compounds revealed preferable drug‐like properties, good oral bioavailability, and safety profiles. Network pharmacology also showed the involvement of SA**1–4** and key targets‐regulated SIRTs in neurodegeneration.

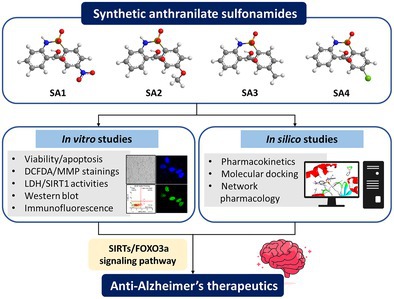

**Abbreviations**
2D: two‐dimensional
3D: three‐dimensional
AA: anthranilic acid
Ac: acetylation
AD: Alzheimer's disease
ADMET: absorption, distribution, metabolism, excretion, and toxicity
ANOVA: one‐way analysis of variance
ATCC: American Type Culture Collection
BAX: Bcl‐2‐associated X‐protein
BBB: blood–brain barrier
BCL‐2: B‐cell lymphoma‐2
BSA: bovine serum albumin
CAT: catalase
CTD: compound–target–disease interaction
DCFDA: 2′,7′‐dichlorodihydrofluorescein diacetate
DMEM: Dulbecco's Modified Eagle Medium
FBS: fetal bovine serum
FOXO3a: forkhead box class O 3a
GO: Gene Ontology
H_2_O_2_
: hydrogen peroxide
HCl: hydrochloric acid
HRP: horseradish peroxidase
IC_50_
: half maximal inhibitory concentration
ICLAC: International Cell Line Authentication Committee
KEGG: Kyoto Encyclopedia of Genes and Genomes
LDH: lactate dehydrogenase
MMP: mitochondrial membrane potential
MTT: 3‐(4,5‐dimethylthiazol‐2‐yl)‐2,5‐diphenyl tetrazolium bromide
NAD^+^
: nicotinamide adenine dinucleotide
ND: neurodegenerative disease
PAGE: polyacrylamide gel electrophoresis
PBS: phosphate‐buffered saline
PDB: Protein Data Bank
PPI: protein–protein interaction
PVDF: polyvinylidene difluoride
ROS: reactive oxygen species
RRID: Research Resource Identifier
RSV: resveratrol
SDS: sodium dodecyl sulfate
SEM: standard error of the mean
SIRTs: sirtuins
SMILES: simplified molecular‐input line‐entry system
SOD2: superoxide dismutase 2
TBST: tris‐buffered saline with Tween‐20
US‐FDA: United States Food and Drug Administration
WHO: World Health Organization

## 1 Introduction

Over the past decades, a global population has gradually become an aging society, which exacerbates a wide range of time‐related functional deterioration, leading to morbidity and mortality‐related non‐communicable neurodegenerative diseases (NDs). Meanwhile, increasing numbers of premature aging and suffering age‐related threats have initiated health and socio‐economic burdens (Gonzales et al. 2022; Kesidou et al. 2023). The World Health Organization (WHO) and the National Health Service are currently prioritizing strategic and technical implementations to provide rapid and effective diagnosis, responsive and safe treatment, as well as alternative prevention (“2023 Alzheimer's disease facts and figures,” 2023; Long et al. 2023). None of the oral therapeutic drugs is currently available for delaying or terminating neuronal damage. However, the passive immunotherapy‐targeted amyloid (i.e., Aducanumab and Lecanemab) with successful phase III clinical trials has been approved for Alzheimer's (AD) remedies by the United States Food and Drug Administration (US‐FDA). On the other hand, small‐molecule neuroprotectants are scarcely ongoing recruited for phase III trials for mild–moderate AD therapies (i.e., anti‐amyloid simufilam, insomniac piromelatine, antidiabetic metformin, caffeine adenosine receptor antagonist, pyrrolo‐pyrimidinone phosphodiesterase 5 inhibitor, xanomeline–trospium muscarinic receptor agonist, as well as targets beyond disease‐modified hydralazine and fosgonimeton) (Cummings, Lee, et al. 2022; Cummings, Montes, et al. 2022; Huang et al. 2023). To counteract this negative phenomenon, computer‐aided drug discovery has been employed to foster effective and safe therapies, reduce cost, and lessen time‐consuming drug development processes (Sun et al. 2022; Wu et al. 2020).

Sulfonamide is one of the well‐recognized pharmacophores in drug design according to its wide‐ranging bioactivities (i.e., antioxidant (Doungsoongnuen et al. 2011; Gocer et al. 2013), anti‐inflammatory (Abdel‐Maksoud et al. 2018; Levin et al. 2002), antimicrobial (Dalloul et al. 2018; Doungsoongnuen et al. 2011), and anticancer (Blank et al. 2011; Powroznik et al. 2016) activities). Anthranilic acid (AA) possesses an anti‐inflammatory effect and is a metabolic intermediate required for cellular nicotinamide adenine dinucleotide phosphate biosynthesis. Accordingly, an attempt has been made towards the design and synthesis of novel sulfonamide‐based compounds as well as repurposing them for new therapeutic applications with a particular focus on those containing anthranilate moieties. Previously, our research group reported a set of bis‐sulfonamide hybrids (Apiraksattayakul et al. 2022), thiazole sulfonamides (Ruankham et al. 2025), and 1,2,3‐triazole‐based sulfonamides (Jongwachirachai et al. 2025) as neuroprotective agents against the neurotoxin 6‐hydroxydopamine‐mediated neuronal cell death in SH‐SY5Y cells. Phenoxythiophene sulfonamide was reported to cross the blood–brain barrier (BBB) and promote neuroprotective effects in a glutamate‐induced oxidative stress model (Pokharel et al. 2022). Additionally, an environmentally friendly synthesis of anthranilate sulfonamides (SA**1–4**) was also reported to display multiple pharmacological properties (i.e., antioxidant, cytotoxic, and antimicrobial activities; Doungsoongnuen et al. 2011). Advances in the emerging interdisciplinary field of network pharmacology‐integrated in silico modeling have led to the assessment of cutting‐edge strategies, allowing for the identification of therapeutic key targets, providing molecular insights into drug interaction, and evaluating the efficient pharmacological interventions in drug discovery and development.

**FIGURE 1:**
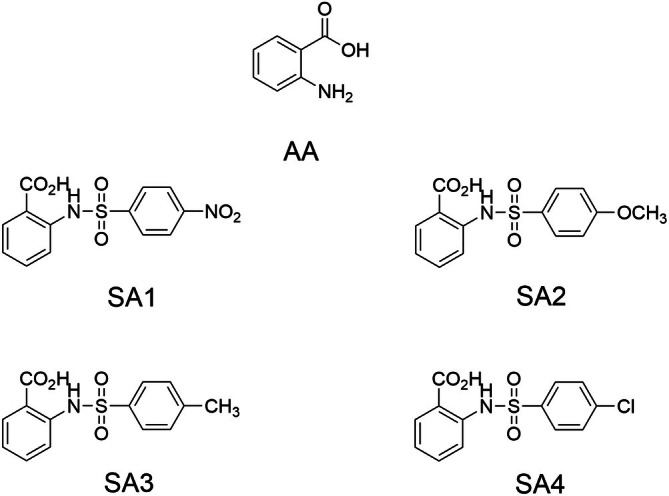
Chemical structures of parent anthranilic acid (AA) and anthranilate sulfonamides (i.e., SA**1**, SA**2**, SA**3**, and SA**4**; Doungsoongnuen et al. 2011).

Herein, the neuroprotective effects of synthetic SA**1–4** (Figure 1) against oxidative injury were investigated through an interdisciplinary approach of in silico and in vitro models. Underlying mechanisms supporting their roles by triggering the sirtuins (SIRTs)/forkhead box class O 3a (FOXO3a) longevity cascade were also explored. Molecular docking was performed to elucidate possible binding modalities of the compounds against the SIRT1 target. Furthermore, in silico pharmacokinetic (ADMET) prediction and network pharmacology were carried out to ensure the potential of compounds for successful development and paved the way for innovative clinical therapeutics, alleviating the burden of NDs.

## 2 Materials and Methods

### 2.1 Investigated Compounds

The hybrids of AA and sulfonamides were synthesized from the general sulfonylation of AA with benzenesulfonyl chlorides in water, followed by purification and characterization using IR and ^1^HNMR analysis (Doungsoongnuen et al. 2011). The custom‐made materials are shared upon reasonable request.

### 2.2 Cell Viability by MTT Assay

Human neuroblastoma SH‐SY5Y cell line was purchased from American Type Culture Collection (RRID: CVCL 0019; ATCC, VA, USA), which is not listed by the International Cell Line Authentication Committee (ICLAC) as commonly misidentified. Although the authentication was not conducted, the cell culture process was recorded from receipt, thawing, culturing, and stock expansion. Briefly, the cells were grown in 96‐well plates containing Dulbecco's Modified Eagle Medium (DMEM) supplemented with 10% fetal bovine serum (FBS) and 1% penicillin/streptomycin antibiotics (Thermo Fisher Scientific Inc., MA, USA) and incubated at 37°C in the condition of 5% CO_2_ humidified incubator for 24 h. After the cells had reached 80% confluence (used between passage number of 40–45), they were subjected to various concentrations (0.1–100 μM) of SA**1–4**, AA, or resveratrol (RSV) for 3 h, followed by exposure to 400 μM hydrogen peroxide (H_2_O_2_) for a further 24 h of incubation. To determine the viable cells, 5 mg/mL of 3‐(4,5‐dimethylthiazol‐2‐yl)‐2,5‐diphenyl tetrazolium bromide (MTT; Molecular Probes, OR, USA) was added to each well, followed by incubation for 3 h under dark conditions. 0.04 N hydrochloric acid (HCl) in isopropanol was applied to solubilize purple crystalline formazan, which was quantified with an absorbance at 570 nm by a microplate reader (RRID: SCR_019743; BioTek Instruments, VT, USA). The half maximal inhibitory concentration (IC_50_) values were calculated using the GraphPad Prism 6 software (RRID: SCR_002798; GraphPad Software Inc., CA, USA).

### 2.3 Lactate Dehydrogenase (LDH) Activity

After the pretreatment with either anthranilate sulfonamides or RSV, the supernatant was transferred to optically clear 96‐well plates. Master reaction mix was set up according to the LDH activity assay kit manufacturer's instructions (#MAK066; Sigma‐Aldrich, MO, USA). The activity of intracellular LDH was spectrophotometrically measured at an absorbance of 450 nm.

### 2.4 Apoptotic Profiles by Flow Cytometry

To determine the apoptotic profiles, the SH‐SY5Y cells were treated as mentioned above. Cell apoptotic assays were performed using Annexin V & Dead Cell Assay Kit (Merck Millipore, Darmstadt, Germany) according to the manufacturer's instructions. Briefly, the SH‐SY5Y cells were harvested and suspended in annexin V/7‐AAD binding buffer, followed by incubation at room temperature for 15 min in the dark condition. The percentage of living, each stage of apoptosis, and dead cells was determined by a Muse cell analyzer (RRID: SCR_020252; Merck Millipore, Darmstadt, Germany).

### 2.5 Intracellular Reactive Oxygen Species (ROS) Levels by Fluorescent DCFDA Staining

In brief, the cells were treated as described above. Subsequently, the cells were stained with 10 μM of 2′,7′‐dichlorodihydrofluorescein diacetate (H_2_DCFDA; Molecular Probes, OR, USA) for 30 min in the dark. The fluorescence signals of ROS formation within the cells were detected by a microplate reader at excitation and emission spectra of 495 nm and 527 nm, respectively, and visualized by a fluorescence microscope (RRID: SCR_018604, Olympus IX70; Olympus, Tokyo, Japan).

### 2.6 Mitochondrial Membrane Potential (MMP) by Fluorescent Rhodamine Staining

MMP was determined by rhodamine 123 fluorescent staining. After the treatment, the cells were washed twice with phosphate‐buffered saline (PBS) and incubated with 10 μM rhodamine 123 dye at 37°C for 30 min in the dark. Emission spectra at 488 nm and excitation spectra at 525 nm were examined by a microplate reader and imaged by a fluorescence microscope.

### 2.7 SIRT1 Enzymatic Activity

Deacetylase activity of SIRT1 was assessed using a commercial fluorometric assay (CS1040; Sigma‐Aldrich, MO, USA). Following the manufacturer's procedure, anthranilate sulfonamide solutions were incubated with the recombinant human SIRT1 enzyme, SIRT1 substrate, and nicotinamide adenine dinucleotide (NAD^+^) in a white flat‐bottom 96‐well plate for screening the cell‐free SIRT1 activity. A SIRT1 activator (RSV) and a SIRT1 inhibitor (sirtinol) were used as positive and negative controls, respectively. The plate was incubated at 37°C for 30 min in a dark atmosphere. The generation of fluorescence intensity at excitation of 340–380 nm and emission of 430–460 nm was monitored using a microplate reader. Moreover, the cellular SIRT1 activity was parallelly determined using lysate of the cells extracted from anthranilate sulfonamide treatment.

### 2.8 Western Blot Analysis

Total protein was extracted with RIPA buffer (Cell Signaling Technology, MA, USA) containing protease inhibitor cocktail (Calbiochem, MA, USA). The concentration of proteins was determined using the Bradford protein assay (Bio‐Rad Laboratories Inc., CA, USA). Protein samples were separated by 12% sodium dodecyl sulfate–polyacrylamide gel electrophoresis (SDS‐PAGE) and then transferred onto polyvinylidene difluoride (PVDF) membranes (GE Healthcare Life Sciences, NJ, USA). The membranes were blocked in tris‐buffered saline containing 0.1% Tween‐20 and 5% nonfat milk (TBST) for 2 h at room temperature. Then, the membranes were incubated with primary antibodies (i.e., SIRT1 (RRID: AB_10999470), SIRT2 (RRID: AB 2636961), SIRT3 (RRID: AB_2188622), FOXO3a (RRID: AB_2636990), BAX (RRID: AB_10557411), BCL‐2 (RRID: AB_2744528), SOD2 (RRID: AB_2636921), CAT (RRID: AB_2798079), and β‐actin (RRID: AB_330288) from Cell Signaling Technology, MA, USA) at 4°C overnight. The probe membranes were washed with TBST, followed by incubation with horseradish peroxidase (HRP) conjugated secondary antibodies at room temperature for 1 h. Bound antibody was detected by Immobilon ECL Ultra Western HRP Substrate (Merck Millipore, Darmstadt, Germany). Protein expression was visualized and quantified using a ChemiDoc MP Imaging System (RRID: SCR_019037) and ImageLab Software (RRID: SCR_014210) (Bio‐Rad Laboratories Inc., CA, USA). The protein expression of the target of interest was normalized with respect to β‐actin housekeeping gene expression. The original gel images are presented in Figure S1.

### 2.9 Immunofluorescence Staining

The immunofluorescence method was performed to detect the presence and localization of SIRT1 protein. Cells were seeded onto a cover slide at a confluence of 80% and then incubated with the anthranilate sulfonamides, followed by 400 μM H_2_O_2_ treatment as previously described. After washing with PBS three times, the cells were fixed with 4% paraformaldehyde for 20 min at room temperature, permeabilized with 1% Triton X‐100, and 1% bovine serum albumin (BSA) to block nonspecific binding targets for 1 h. Primary mouse polyclonal antibody against SIRT1 (1:1000 dilution) was added to the coverslips at 4°C overnight. Alexa Fluor 488 conjugated with anti‐mouse IgG (1:500 dilution; RRID: AB_10694704; Cell Signaling Technology, MA, USA) was used to detect primary antibodies, followed by counterstaining with DAPI for 10 min. The expression and localization of SIRT1 in the SH‐SY5Y cells were then imaged using a confocal microscope (RRID: SCR_014215, Olympus FluoView FV1000; Olympus, Tokyo, Japan).

### 2.10 Molecular Docking Study

A molecular docking study was performed to reveal possible binding modes of the compounds against the SIRT1 target using AutoDock 4.2.6 software. The three‐dimensional (3D) structure of SIRT1 protein was obtained from the Protein Data Bank (PDB ID: 5BTR) (Cao et al. 2015) and further refined by removing water molecules and co‐crystallized ligand, RSV. Prior to subsequent docking steps, the protein structure was prepared by the addition of polar hydrogens and Gasteiger charges using the implemented AutoDock tools 1.5.7 (RRID: SCR_026401). The chemical structures of the investigated compounds were drawn and minimized force field, using ChemOffice 2020 (PerkinElmer Informatics Inc., CT, USA). The molecular grid box was generated by the AutoGrid program to completely cover the centered active site of the SIRT1 protein with a spacing of 0.375 Å and grid dimensions of −23.315 × 65.89 × 14.723 Å^3^ in the *x*, *y*, and *z* dimensions, respectively. Redocking of the co‐crystalized ligand was performed to validate the docking protocol. The Lamarckian genetic algorithm was employed for 100 docking simulations (Morris et al. 1998). The lowest binding energy conformation of each compound was chosen for the model analysis. The 2D/3D interactions and the active site residues were generated by Discovery Studio Visualizer 2021 (RRID: SCR_008398; BIOVIA, Dassault Systèmes, CA, USA).

### 2.11 ADMET Prediction

2D chemical structures of anthranilate sulfonamides and parent AA were drawn and converted to a simplified molecular‐input line‐entry system (SMILES) format using ChemOffice 2020 software. Pharmacokinetic parameters regarding absorption, distribution, metabolism, excretion, and toxicity (ADMET) were predicted using three web‐based tools, including SwissADME (http://www.swissadme.ch/) (Daina et al. 2017), pkCSM (http://biosig.unimelb.edu.au/) (Pires et al. 2015), and ADMETLab 2.0 (https://admetmesh.scbdd.com/) (Xiong et al. 2021), to reassure the precision of different algorithmic elucidation.

### 2.12 Network Pharmacology Analysis

The SMILES format of anthranilate sulfonamides was imported into the online databases such as SwissTargetPrediction (RRID: SCR_023756, https://www.swisstargetprediction.ch/), SuperPRED (RRID: SCR_002691, https://prediction.charite.de/subpages/target_prediction.php), and SEA Search Server (https://sea.bkslab.org/), to predict the potential targets related to multiple diseases in human. Target‐related NDs were obtained from the DisGeNET (RRID: SCR_006178, https://www.disgenet.org/) and GeneCards (RRID: SCR_002773, https://www.genecards.org) databases by using the related terms of “Human,” “Neurodegenerative disorders,” “Neurodegenerative diseases,” and “ND.” The overlapping targets of anthranilate sulfonamides and the NDs, by a Venn diagram, were analyzed through the STRING database version 12.0 (RRID: SCR_005223, https://string‐db.org/) with the medium confidence level of 0.4 scores, and further constructed the network of “compound–target–disease interaction (CTD)” by the Cytoscape 3.10.1. software (RRID: SCR_025009, https://cytoscape.org/). Employing the “protein–protein interaction (PPI)” from Gene Ontology (GO) enrichment and Kyoto Encyclopedia of Genes and Genomes (KEGG) pathway, the top 20 core targets and mechanisms of anthranilate sulfonamides were identified by the ShinyGO 0.82 (RRID: SCR_019213, http://bioinformatics.sdstate.edu/go/).

### 2.13 Statistical Analysis

Statistical calculations were performed using GraphPad Prism 6 software. The sample size for each experiment was not pre‐determined by formal calculation; instead, the setups were based on previous similar experimental designs (Gay et al. 2018; Ruankham et al. 2021). No blinding and outlier experiments were performed. The significant differences between three or more groups with normal distribution, as determined by the visual histogram, were analyzed using parametric one‐way analysis of variance (ANOVA) followed by a Tukey–Kramer post hoc test. All values are presented as mean ± standard error of the mean (SEM) values from three biological replicates (independent passages or experimental runs). *p < 0.05 and ^#^ *p* < 0.05 are considered statistically significant compared with the untreated cells and H_2_O_2_ group, respectively.

## 3 Results

### 3.1 Anthranilate Sulfonamides Inhibit Cytotoxicity Against H_2_O_2_ ‐Induced Human SH‐SY5Y Cells

**FIGURE 2:**
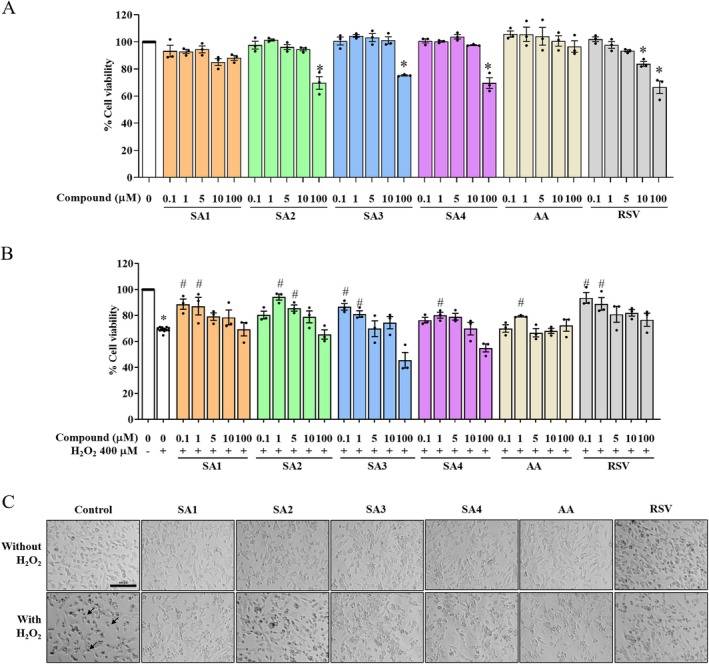
Anthranilate sulfonamides do not induce cytotoxicity at 1 μM. (A) The effects of SA**1–4** on the viability of human neuroblastoma SH‐SY5Y cells were assessed by MTT assay. (B) The synthetic SA**1–4** exerted viable rates against exogenous hydrogen peroxide (H_2_O_2_) exposure. In the bar graphs, each dot represents a single measurement and mean ± SEM of the quantification data from *n* = 3 independent passages. Statistical annotations of *p < 0.05 and ^#^ *p* < 0.05 were significantly compared with the untreated cells and the H_2_O_2_ group, respectively. (C) The morphological changes of SA**1–4** pretreatments were captured at ×20 magnification (Bar = 500 μm in length). Arrows defined the morphologically apoptotic‐like behaviors.

To investigate the promising capacity of SA**1–4** whether they provide neuroprotective effects on oxidative stress‐mediated neurodegeneration due to exogenous H_2_O_2_, human neuroblastoma SH‐SY5Y cells derived from the sympathetic nervous system were used as a representative of the in vitro catecholaminergic model for neurodegenerative studies. The cell viability of compounds SA**1–4** and the parent AA pretreatment was assessed using an MTT assay. Various concentrations of the compounds (varying from 0.1, 1, 5, 10, and 100 μM) were screened for the optimum concentration. A one‐way ANOVA following normal distribution showed no significant cytotoxic effect at 0.1–100 μM of SA**1** and AA, whereas the viability of the cells exposed to 100 μM SA**2**, SA**3**, and SA**4** was significantly decreased compared with the control (Figure 2A, *F* (30, 77) = 18.230, *p* < 0.0001). The relative cytotoxicity of SA**1** and AA showed IC_50_ values greater than 500 μM after 24 h of treatment, followed by SA**3**, SA**4**, SA**2**, and RSV (325.0, 239.6, 226.3, and 172.2 μM), respectively. This was concurrent with the concentration‐dependent biphasic behaviors of a well‐known antioxidant RSV (Ruankham et al. 2023; Salehi et al. 2018).

H_2_O_2_ has been extensively employed in in vitro models as an aggressive oxidizing agent due to its effect via disturbing intracellular antioxidant defenses associated with age‐related neurodegenerative conditions. To imitate oxidative stress‐induced neurodegeneration, 400 μM of exogenous H_2_O_2_ was chosen as described in the previous studies (Gay et al. 2018; Ruankham et al. 2021). Human neuronal SH‐SY5Y cells were pretreated with desired concentrations of SA**1–4** and AA (0.1, 1, 5, 10, and 100 μM) for 3 h prior to encountering H_2_O_2_ 400 μM for further incubation of 24 h. The MTT assay documented that the viable percentage of cells exposed to H_2_O_2_ at the indicated concentrations was markedly decreased to less than 30.72% of the unexposed group. Surprisingly, the data were normally distributed, and a one‐way ANOVA revealed a significant treatment effect (*F* (31, 82) = 14.690, *p* < 0.0001). Pretreatment of SH‐SY5Y cells with 0.1–5 μM of SA**1–4**, AA, and RSV significantly increased the viable rate of cells compared with the H_2_O_2_‐exposed cells (Figure 2B). Similar results were supported by microscopic observation of SA**1–4** and AA pretreatment (Figure 2C). Morphological changes of H_2_O_2_‐treated cells were evident as shrinkage, short neurites, and small vesicular bodies with respect to the control. As expected, SA**1–4** and AA themselves did not influence the apoptotic effects compared with the untreated group. Pretreatment of SA**1–4** and AA improved apoptosis‐appearing neurons compared with the H_2_O_2_‐treated group, which was correlated with the RSV results. These suggested that the compounds SA**1–4** at 1 μM were not toxic to the cells and represented predominant protective effects; therefore, the concentration of 1 μM was selected for further experiments.

### 3.2 Anthranilate Sulfonamides Enhance Diverse Biological Activities Against ROS Generation

**FIGURE 3:**
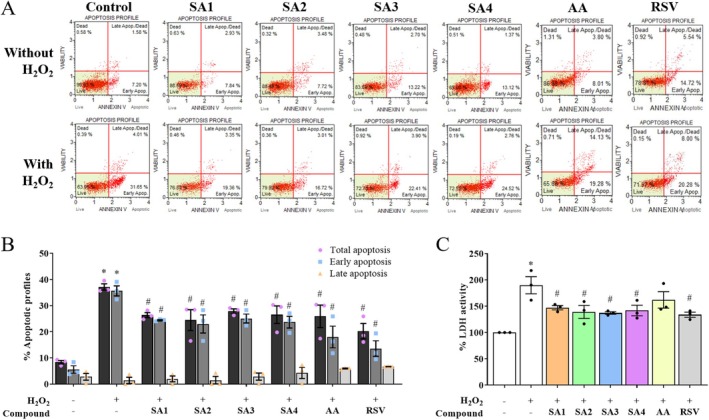
Anthranilate sulfonamides maintain anti‐apoptotic properties in hydrogen peroxide (H_2_O_2_)‐treated SH‐SY5Y cells. (A) The scattergram of SA**1**–**4** was characterized in various manners: viable (lower left), early apoptosis (lower right), late apoptosis (upper right), and dead cells (upper left). (B) The percentage of apoptotic profiles was quantified by flow cytometry. (C) The percentage of lactate dehydrogenase (LDH) activity was quantified by LDH enzymatic assay. In the bar graphs, each dot represents a single measurement and mean ± SEM of the quantification data from *n* = 3 independent passages. Statistical annotations of *p < 0.05 and ^#^ *p* < 0.05 were significantly compared with the untreated cells and the H_2_O_2_ group, respectively.

Based on the abilities of compounds to enhance cell survival rate, the effect of compound pretreatment and anti‐apoptotic activities was subsequently explored by annexin V/7‐AAD double staining. Such a staining technique differentiated four cell populations, including live stage, early apoptosis, late apoptosis, and cell death, as represented by scatter plots (Figure 3A). ROS production in response to H_2_O_2_ treatment affected several apoptotic population, which significantly increased by approximately 40% compared with the control. The data were normally distributed, followed by a one‐way ANOVA (*F* (14, 48) = 6.047, *p* < 0.0001). There was no significant difference in the late‐stage apoptosis for the cells exposed to H_2_O_2_ in comparison to the control. Similarly, the compounds (SA**1–4** and parent AA) themselves did not induce apoptosis in SH‐SY5Y models (Figure 3A, upper panel). However, pretreatment with SA**1–4** and AA markedly decreased the apoptotic amount of early stage and total apoptosis due to H_2_O_2_ treatment compared with the H_2_O_2_ alone in a well‐concurrent manner to that of the antioxidant RSV (Figure 3A, lower panel and 3B).

**FIGURE 4:**
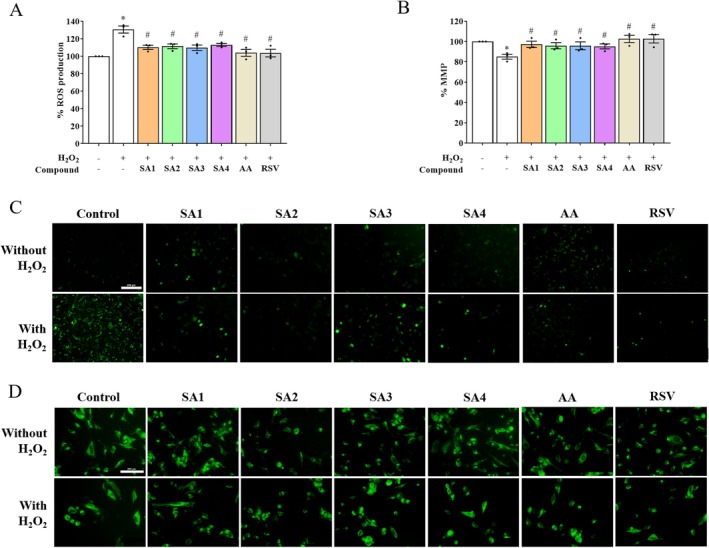
Anthranilate sulfonamides maintain antioxidant properties against hydrogen peroxide (H_2_O_2_)‐treated SH‐SY5Y cells. The percentages of (A) reactive oxygen species (ROS) production and (B) mitochondrial membrane potential (MMP) signal were quantified by fluorescent DCFDA and rhodamine stainings, respectively. In the bar graphs, each dot represents a single measurement and mean ± SEM of the quantification data from *n* = 3 independent passages. Statistical annotations of *p < 0.05 and ^#^ *p* < 0.05 were significantly compared with the untreated cells and the H_2_O_2_ group, respectively. The immunofluorescent detections of (C) ROS generation and (D) MMP of SA**1**–**4** pretreatments against oxidative stress were captured at ×20 magnification (Bar = 500 μm in length).

To support the protective effects, the normal distribution of the leakage of LDH enzyme (Figure 3C) and ROS generation (Figure 4A) was analyzed using one‐way ANOVA (*F* (7, 16) = 6.440, *p* = 0.0010 and *F* (7, 16) = 9.203, *p* = 0.0001, respectively). Treating the human SH‐SY5Y cell line with H_2_O_2_ at a constant level of 400 μM increased the leakage of LDH enzyme and intracellular ROS production up to 189.76% and 130.58%, respectively, compared with those of the unexposed cells. Pretreatment with 1 μM of SA**1–4**, followed by 400 μM H_2_O_2_ treatment, significantly suppressed LDH cytotoxicity, whereas pretreatment with AA showed no significant suppression. In contrast, pretreatments with SA**1–4** and AA were shown to fully restore ROS balance, with a comparable effect to that of the RSV, when compared with the H_2_O_2_ exposure. The intensity of green fluorescent staining of DCFDA was captured and was significantly higher in H_2_O_2_‐induced ROS generation, whereas pretreatment with SA**1–4** and AA abolished the intensity of ROS signal (Figure 4C). The mitochondrial damage of SH‐SY5Y cells due to H_2_O_2_ was also examined by fluorescent rhodamine staining (Figure 4B). A one‐way ANOVA of the normally distributed data revealed a significant treatment effect (*F* (7, 16) = 3.322, *p* = 0.0222). As shown in Figure 4D, the results were contrary to the observation on ROS generation, in which exposure to H_2_O_2_ led to a decline of MMP. This is one of the indicators for tracking the mitochondrial status, by 85.05%. However, the MMP levels were maintained in a similar manner to that of the RSV, when the cells were pretreated with compounds (SA**1**–**4** and AA). These suggested that the compounds SA**1**–**4** may modulate mitochondrial function and suppress an early stage of apoptosis.

### 3.3 Potential Role of Sirtuins Contributes to Anthranilate Sulfonamides‐Activated Neuroprotection

**FIGURE 5:**
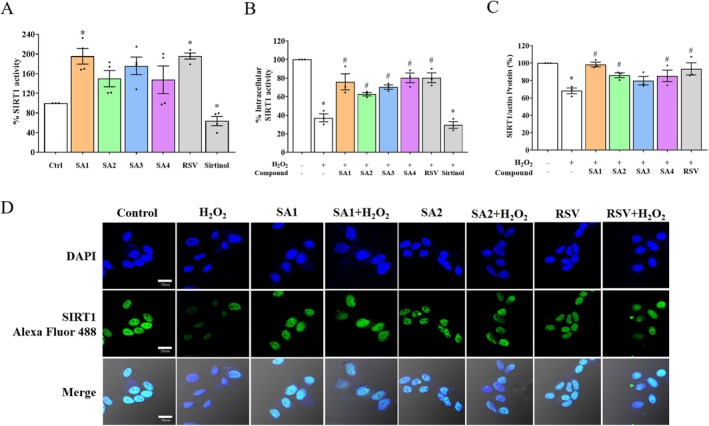
Anthranilate sulfonamides protect neuronal cells through the sirtuin 1 (SIRT1) longevity. The SIRT1 activity was determined by (A) cell‐free and (B) SA**1**–**4**‐pretreated cells. (C) The protein expression of SIRT1 was evaluated by Western blot. In the bar graphs, each dot represents a single measurement and mean ± SEM of the quantification data from *n* = 3 independent passages. Statistical annotations of *p < 0.05 and ^#^ *p* < 0.05 were significantly compared with the untreated cells and the H_2_O_2_ group, respectively. (D) The cellular localization of SIRT1 was fluorescently visualized at ×60 magnification (Bar = 20 μm in length).

To investigate the role of SA**1**–**4** on SIRTs‐induced neuronal survival signaling, cell‐free SIRT1 activity was assessed alongside the cellular levels of SIRT1 activity‐mediated neuronal cells (Bononi et al. 2022; Flori et al. 2021). For datasets with normal distribution, a one‐way ANOVA demonstrated that the recombinant SIRT1 deacetylase enzyme without any activation was set as the control at a percentage of 100, as shown in Figure 5A (*F* (6, 20) = 8.887, *p* < 0.0001). RSV and sirtinol at a concentration of 1 μM were used as the positive and negative substances, respectively. The result showed that RSV predominantly activated SIRT1 activity up to 196.02%, whereas sirtinol markedly suppressed the activity by approximately 63.72%. Among all investigated compounds, SIRT1 deacetylase activity tended to be increased, but was significantly enhanced by SA**1** (195.65%) in a similar manner to the RSV. The activation of SIRT1‐deacetylated enzyme by SA**1**–**4** was further speculated on their cellular potencies of activation (Figure 5B, *F* (7, 16) = 26.130, *p* < 0.0001). The neuronal cells exposed to H_2_O_2_ dramatically mitigated the activity of the SIRT1 enzyme (37.24%) with a comparable effect to that of sirtinol (29.58%) when compared with the control. All investigated SA**1**–**4** and RSV, except for the parent AA, significantly increased the intracellular SIRT1 activity in the H_2_O_2_‐induced cells. The increase in SIRT1 activity is known to induce the production of SIRT1; thus, protein expression of SIRT1 was determined to support deacetylase activity in response to the compound's pretreatment (Figure 5C). The data were normally distributed, followed by a one‐way ANOVA (*F* (6, 14) = 6.133, *p* = 0.0025). Treatment with H_2_O_2_ significantly decreased SIRT1 protein compared with that of the control, whereas pretreatment with SA**1**, **2**, and **4** caused an increase in SIRT1 protein expression in the H_2_O_2_‐induced cells with a similar effect to that of RSV. To determine whether these compounds could facilitate SIRT1 nuclear translocation, the SH‐SY5Y cells were co‐stained with bright blue DAPI and green SIRT1‐Alexa Fluor488‐specific antibody. Immunostaining revealed that SIRT1 was mainly localized in the nucleus, and the green fluorescent signal of SIRT1 was hindered by H_2_O_2_ exposure. As expected, both SA**1** and **2** pretreatments did not affect the fluorescence intensity of SIRT1 inside the nucleus compared with the control. Both compounds visibly recovered the SIRT1 signals against H_2_O_2_‐intoxicated oxidative injury, like the RSV (Figure 5D).

**FIGURE 6:**
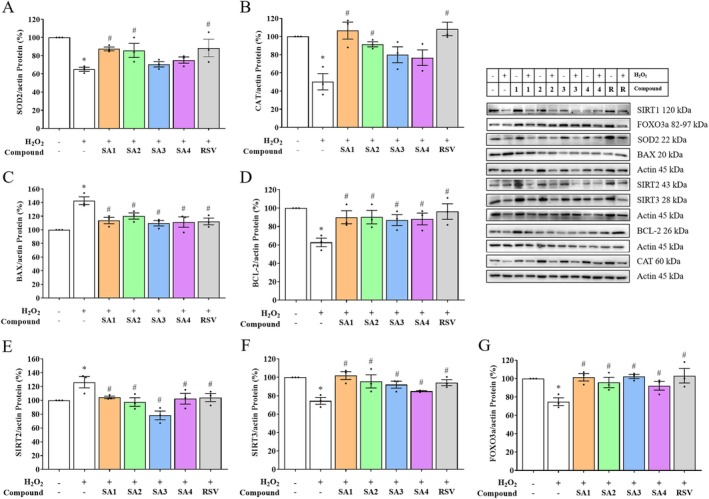
Anthranilate sulfonamides modulate the downstream cascade of neuroprotective sirtuins (SIRTs) family in hydrogen peroxide (H_2_O_2_)‐treated SH‐SY5Y cells. (A) superoxide dismutase 2 (SOD2), (B) catalase (CAT), (C) pro‐apoptotic Bcl‐2‐associated X‐protein (BAX), (D) anti‐apoptotic B‐cell lymphoma‐2 (BCL‐2), (E) sirtuin 2 (SIRT2), (F) sirtuin 3 (SIRT3), and (G) forkhead box class O 3a (FOXO3a) protein expressions of SA**1**–**4** pretreatments induced by H_2_O_2_ exposure were evaluated by Western blot analysis. In the bar graphs, each dot represents a single measurement and mean ± SEM of the quantification data from *n* = 3 independent passages. Statistical annotations of *p < 0.05 and ^#^ *p* < 0.05 were significantly compared with the untreated cells and the H_2_O_2_ group, respectively.

Since the sirtuin family acts as a longevity factor and the investigated compounds are capable of modulating SIRT1, sirtuin members like SIRT2 and SIRT3 were further explored for their underlying mechanisms. When the cells underwent the H_2_O_2_‐induced oxidative event, the SIRT2 protein expression (Figure 6E, *F* (6, 14) = 5.639, *p* = 0.0037) significantly increased up to 126.29%, whereas that of the SIRT3 was observed in the opposite direction at a decreased percentage of 68.17 (Figure 6F, *F* (6, 14) = 5.776, *p* < 0.0033) compared with the untreated group. Interestingly, the protein expressions of SIRT2 and SIRT3 in the SA**1**–**4**‐pretreated group were significantly maintained against H_2_O_2_ exposure, consistent with that of RSV. These led to further mitochondrial protection through the expression of FOXO3a (Jęśko and Strosznajder 2016), a downstream regulator of neuroprotective SIRTs signaling pathways sensitive to oxidative stress and apoptosis. As shown in Figure 6G (*F* (6, 14) = 4.451, *p* = 0.0100), the protein expression of FOXO3a in the H_2_O_2_‐treated group was lower (74.95%) than the control group. Particularly, pretreatment with SA**1**–**4** significantly increased the expression levels of FOXO3a compared with the non‐pretreated H_2_O_2_ exposure. These data suggested that the FOXO3a mediated‐oxidative stress response can be precisely deacetylated by SIRT1, SIRT2, and SIRT3, which are NAD^+^‐dependent deacetylases, to increase longevity.

To determine the SIRTs downstream targets of SA**1**–**4** on antioxidant signaling pathways, the protein expressions of endogenous antioxidant enzymes, including superoxide dismutase (SOD2; Figure 6A, *F* (6, 14) = 5.654, *p* = 0.0036) and catalase (CAT; Figure 6B, *F* (6, 14) = 7.799, *p* = 0.0008), were determined. Western blotting analysis indicated that both antioxidant enzymes were significantly downregulated by H_2_O_2_. Interestingly, SA**1** and SA**2** pretreatments rescued the expression levels of SOD2 and CAT compared with that of the H_2_O_2_‐exposed cells. These were consistent with those observed in the RSV pretreatment. Although SA**3** and SA**4** decreased levels of ROS production, both compounds were incapable of restoring the protein expressions of SOD2 and CAT. This contradiction could be due to the isoform of mitochondrial matrix SOD2 (Doungsoongnuen et al. 2011). Taken together, pretreatment with SA**1**–**4** restored antioxidant status, particularly SA**1** and SA**2**, which could mediate antioxidant defense via regulating the expressions of endogenous antioxidant enzymes (i.e., SOD2 and CAT). These suggested that they may modulate mitochondrial function and suppress an early stage of apoptosis.

The result also revealed that the expression of pro‐apoptotic BAX protein was prominently increased up to 142.63% during the H_2_O_2_ exposure (Figure 6C, *F* (5, 12) = 8.444, *p* = 0.0013), whereas that of the anti‐apoptotic BCL‐2 (Figure 6D, *F* (5, 12) = 4.638, *p* = 0.0137) was dramatically inhibited by approximately 62.75% compared with the control. This implies that the cells undergo apoptotic cascades, which can affect their survival. In accordance with the RSV‐pretreated group, pretreatment with SA**1**–**4** suppressed BAX but increased BCL‐2 protein expression compared with the group solely exposed to H_2_O_2_. The findings indicated that these compounds could modulate the levels of pro/anti‐apoptotic proteins, supporting their observed inhibitory effects against apoptosis‐mediated NDs, which are accompanied by the recent study of AA‐memantine cotreatment in the amyloid‐treated neuronal hippocampus (Ovey and Naziroglu 2021).

### 3.4 Anthranilate Sulfonamides Accommodate an Active Site of SIRT1 Protein

**FIGURE 7:**
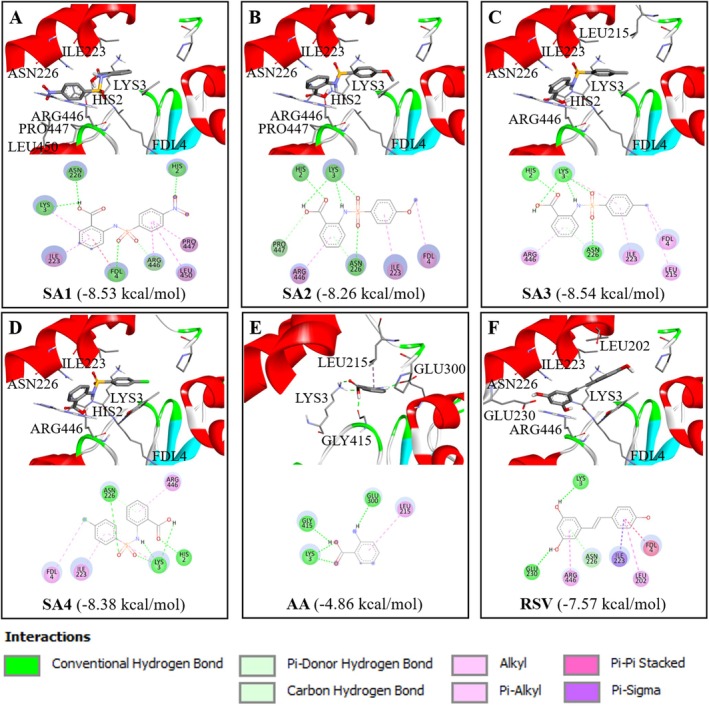
Two (2D) and three (3D)‐dimensional binding poses of anthranilate sulfonamides docked in the active binding site of sirtuin 1 (SIRT1) (PDB: 5BTR). The interactions of SIRT1 protein and ligand (i.e., (A) SA**1**, (B) SA**2**, (C) SA**3**, (D) SA**4**, (E) anthranilic acid (AA), and (F) resveratrol (RSV)) were predicted by molecular docking. The annotation is represented as bond interactions.

The binding behavior of investigated compounds (SA**1**–**4**) within the binding site of SIRT1 longevity protein was explored by molecular docking approach. Crystal structure of SIRT1 in complex with an RSV and an artificial p53‐complexed 7‐amino‐4‐methylcoumarin (AMC) substrate (PDB ID: 5BTR) was used as a target protein. The redocking of the co‐crystalized ligand (RSV) was performed to verify the reliability of the docking protocol, and a root mean square deviation (RMSD) was calculated. RMSD less than 2.0 Å is well‐known for indicating acceptable accuracy (Ramírez and Caballero 2018). Herein, redocking results provided the calculated RMSD value of 0.57 Å, indicating the reliability of the simulation (Ramírez and Caballero 2018). The same protocol and parameters were further applied to simulate the binding of studied compounds (SA**1**–**4** and parent AA) against the active binding site of SIRT1, and their molecular interactions were investigated (Figure 7). The docking simulations revealed that all compounds (SA**1**–**4** and parent AA) could occupy well within the identical binding site of the RSV on the target SIRT1. Estimated binding free energy, binding interactions, and key amino acids are summarized in Table S1. It was revealed that the synthetic derivatives (SA**1**–**4**) provided better binding energy values ranging from −8.26 to −8.54 kcal/mol than that of the parent AA (−4.86 kcal/mol), as well as a reference RSV (−7.57 kcal/mol). It was shown that the compounds (SA**1**–**4** and AA) bind against the SIRT1 target via various types of interactions (i.e., hydrogen bonds, π‐type interactions, and alkyl bonds). RSV is a well‐known SIRT1 activator; therefore, it is used as a reference for comparative analysis of binding interactions. All studied compounds, except for the parent AA, shared common key interacting amino acids with those of the RSV (i.e., ILE223 and ASN226 located on the N‐terminal domain, and ARG446 on the catalytic domain of SIRT1).

### 3.5 Network Pharmacology Reveals Possible Targets in Anthranilate Sulfonamides‐Associated Neurodegeneration

**FIGURE 8:**
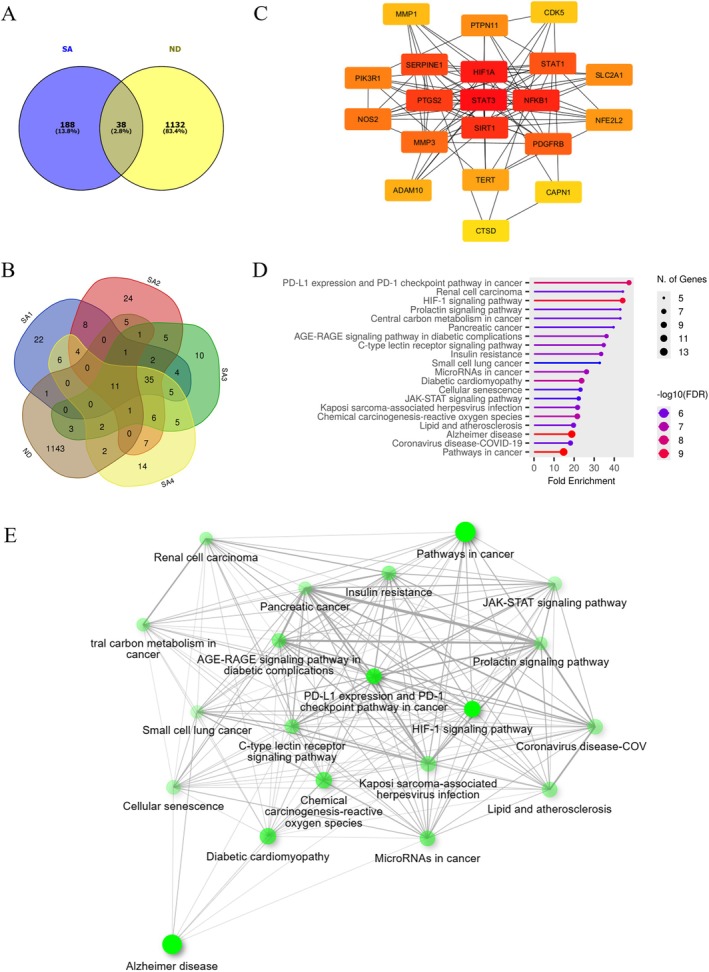
Network pharmacology of anthranilate sulfonamides and neurodegenerative diseases (NDs). (A) Overlapping target proteins and compounds by a Venn diagram. (B) Targets identified by each compound (SA**1**–**4**). (C) Protein–protein interaction (PPI) network of the top 20 targets, with key targets identified through centrality analyses. (D) Top 20 and (E) network diagram of core Gene Ontology (GO) terms ranked by enrichment analysis in several biological processes.

To gain an in‐depth mechanism of anthranilate sulfonamides in NDs, network pharmacology analysis was conducted. A total of 226 anthranilate sulfonamides‐targeted network was retrieved from the SwissTargetPrediction, SuperPRED, and SEA databases, with score > 0, probability ≥ 60, and *Z*‐score ≥ 50, respectively. Subsequently, the targets for NDs were collected by integrating data from the DisGeNET and GeneCards databases, with a relevance score ≥ 20 for screening conditions. After combining all data and removing ineligible and duplicate targets, 1170 unique targets were identified. Comparison of these genes with compounds‐derived neurodegenerative targets using a Venny diagram revealed 38 common intersection targets (Figure 8A), which were visualized in the specific CTD network as potentially relevant to neuroprotection in humans of each SA**1**–**4** in Figure 8B. Following the identification of 38 potential targets, a PPI network linked to SA**1**–**4**‐related NDs was constructed using the STRING database and classified by the Cytoscape platform (Figure 8C). The color intensity of the nodes correlates with their calculated degree, with more intense coloring indicating higher degree values. Edges represent the protein interactions. Additionally, a range of complementary topological algorithms from the CytoNCA and cytoHubba plugins, that is degree, closeness, and betweenness, were utilized to identify the top 20 highly connected targets within the PPI network, as listed in Table S2. Target genes were significantly enriched in cellular response to inflammation (i.e., STAT3, NFKB1, PTGS2, STAT1, and NOS2), hypoxia (i.e., HIF1A and NFE2L2), longevity (i.e., SIRT1 and TERT), cell proliferation/apoptosis (i.e., PDGFRB, PTPN11, SERPINE1, CDK5, and CAPN1), collagen degradation (i.e., MMP3 and MMP1), glucose uptake (i.e., PIK3R1 and SLC2A1), and APP processing (i.e., ADAM10 and CTSD).

GO pathway enrichment analysis of the 38 selected target genes identified the top 20 enriched pathways, which were ranked based on gene amounts and fold enrichment (Figure 8D). The size of each ball represents the number of genes, whereas the color saturation corresponds to false discovery rate (FDR) values, with smaller values indicating greater enrichment significance. Among them, “Pathways in cancer” exhibited the highest gene enrichment (13 genes), followed by “Alzheimer's disease” (12 genes), “HIF‐1 signaling pathway” (8 genes), “Diabetic cardiomyopathy” (8 genes), “Kaposi sarcoma‐associated herpesvirus infection” (7 genes), “Lipid and atherosclerosis” (7 genes), and “Coronavirus disease‐COVID‐19” (7 genes). As shown in Figure 8E, the co‐expression target genes were significantly involved in several biological processes and regulated by many transcription factors‐related GO pathway enrichment. Physically, the abovementioned interactions imply the protein collaboration of cellular functions encoded by these genes.

**FIGURE 9:**
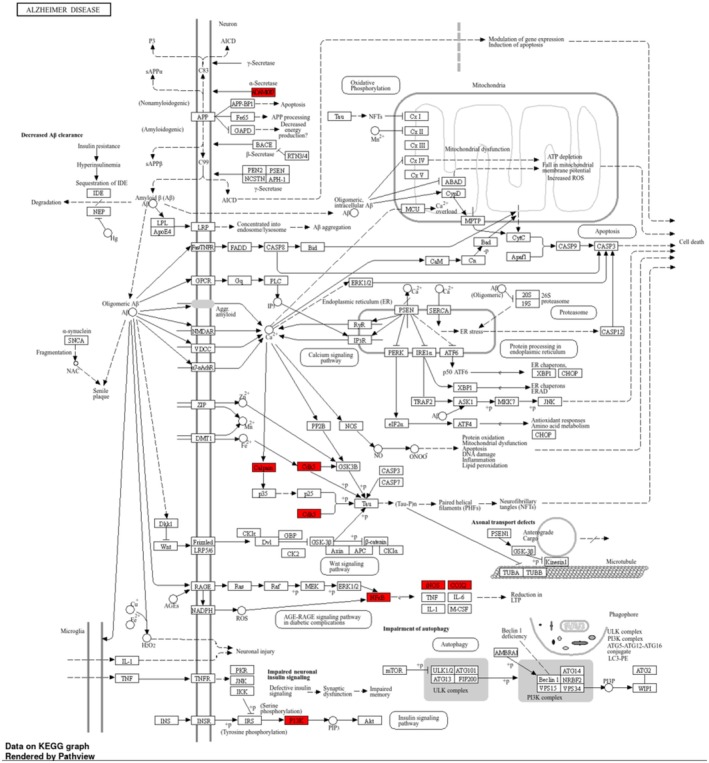
Key protein interrelationships of anthranilate sulfonamides in Alzheimer's disease (AD).

Specifically, the AD signaling pathway by KEGG pathway enrichment analysis was visualized in Figure 9. The key targets are represented as red boxes in the KEGG signaling pathway enrichment. These indicate the distribution of potential target genes within 5 major pathways, including non‐amyloidogenic cascade of ADAM10 (Khezri et al. 2023), tau phosphorylation by CDK5 (Kimura et al. 2014), CAPN1 in calcium homeostasis (Mahaman et al. 2019), PI3K in insulin‐mediated glucose uptake (Gabbouj et al. 2019), and advanced glycation end products (AGEs) and their receptor RAGE activating downstreams of NFKB, iNOS, and COX2 (Bhattacharya et al. 2023), which are concurrent with the neuroprotection by the SA**1**–**4**‐regulated SIRTs. These findings support the pivotal neuroprotective roles of anthranilate sulfonamides in regulating multiple signaling pathways related to oxidative stress, apoptosis, and inflammation, especially in AD.

### 3.6 Anthranilate Sulfonamides Possess Drug‐Likeness

Several open‐access platforms of in silico ADMET prediction were employed, including SwissADME, pkCSM, and ADMETLab, which have been documented as an excellent coverage of the larger in vivo physicochemical and pharmacokinetic parameters (Dulsat et al. 2023). The virtual prediction of prospective therapeutic candidates is represented in Table S3. In regard to the most common Lipinski's rule applied to pharmaceutical regulations, molecular weight less than 500, hydrogen‐bond donors less than 5, hydrogen‐bond acceptors less than 10, and lipophilicity less than 5 were generally considered for drug‐like compounds (Lipinski 2005, 2016). Along with the rules of Lipinski, additional parameters supported by Veber et al. (2002) (i.e., polar surface area not greater than 140 Å and rotatable bond not greater than 10) and Ghose et al.'s (1999) rules (i.e., molar refractivity in a range of 40–130 and total number of atoms between 20 and 70) were also analyzed. The prediction showed that all compounds (SA**1**–**4**) passed the criteria without any violations except for the parent AA.

Regarding the predicted ADMET parameters, all compounds have moderate to high aqueous solubility, high intestinal absorption, and high skin permeability, as well as moderate to high human Caco‐2 cell permeability. This suggested that they are potentially formulated for oral administration. Considering the distribution parameters, BBB and central nervous system (CNS) values were all positive, indicating the efficacy of these compounds to distribute into the target site in the CNS. The predicted volume of distribution (VDss) is low, with human fraction unbound in the plasma ranging from 0.16 to 0.517 log L/kg, suggesting their low distribution into the tissues. Neither of the ligands acted as CYP2D6, CYP3A4, and renal OCT2 substrates nor CYPs (1A2, 2C19, 2C9, 2D6, and 3A4) inhibitors. This suggested that these compounds may be metabolized via other alternative paths and display low potential to induce drug–drug or food–drug interaction.

Total clearance was computed between 0.021 and 0.587 log mL/min/kg. None of the investigated derivatives has a positive AMES carcinogenic toxicity, and most of them are non‐hepatotoxic agents (Table S4). These compounds are considered relatively safe as they were predicted to be non‐carcinogenic agents towards human cholangiocarcinoma HuCCA‐1, lung epithelial A549, and hepatoblastoma HepG2 cells (Doungsoongnuen et al. 2011). Thus, all anthranilate sulfonamide derivatives exhibited preferable drug‐likeness properties together with reasonable CNS permeability and oral administration potential.

## 4 Discussion

NDs are characterized by the coexistence of complexity in clinical/neuropathological features. Because of the global aging evolution, the burden of chronic NDs globally affected approximately 55 million dementia elderly in 2022 and is expected to be 139 million in 2050 (Gonzales et al. 2022; Kesidou et al. 2023). On top of that, symptomatic treatments are temporarily applied to treat common symptoms without addressing the pathophysiological factors of the diseases (Cummings, Lee, et al. 2022; Cummings, Montes, et al. 2022; Huang et al. 2023). Accordingly, the discovery of novel disease‐modifying agents is essential for effective disease management. It was documented that 90% of the clinical failures and approval rejections of developing therapeutics have been attributed to insufficient preclinical models, non‐relevant CNS targets, limited neuropathophysiological/neurophenotypic understandings, along with inefficient drug‐like properties and undesirable toxicity (Kim et al. 2022; Sun et al. 2022). This indicates that the development of novel drug candidates is definitely challenging in the pharmaceutical industry; thereby, computational approaches have been widely used to facilitate the process (i.e., minimize time‐consuming, extravagant human and material resources, and overpriced investment) (Sun et al. 2022; Wu et al. 2020).

AA (Prasher and Sharma 2021) and sulfonamide (Culletta et al. 2023) pharmacophores are attractive scaffolds for drug design. Structural modifications on these scaffolds give compounds with high structural diversity for widespread pharmaceutical applications. Examples were shown by the modifications of clinically available drugs by adding the sulfonamide group to their molecules to provide new derivatives for drug repurposing. Regarding the anti‐aging property of NAD^+^‐dependent SIRTs deacetylases in mammalian species, modulation of SIRT1 activity has been reported to be the most well‐characterized beneficial strategy for various biological processes during metabolism and homeostasis, in which its modulations affect regulations of several transcription factors and downstream targets relating to redox‐based signaling cascades (Jesko et al. 2017; Polito et al. 2017; Pukhalskaia et al. 2022).

In this study, the neuroprotective effect and potential underlying mechanisms of anthranilate sulfonamides were elucidated on oxidative damage‐related NDs. The findings revealed that the synthetic derivatives (SA**1**–**4**) could better mimic the SIRT1 binding behavior of the RSV due to the presence of two terminal benzene rings and a sulfonamide group in their molecules when compared with the parent AA. These two benzene rings could mimic the core skeleton of the RSV by forming the pi‐interactions with ILE223 and ARG446 residues of SIRT1, whereas the sulfonyl oxygen atom of the sulfonamide linker (SA**2**, SA**3**, and SA**4**) or carboxylic group of the SA**1** plays key roles in hydrogen bond formation with ASN226 residue. Notably, this addition of conventional hydrogen bond formation with ASN226 was not observed for the binding of RSV and could contribute to the better binding affinity of the SA**1**–**4** beyond the RSV. Consequently, it may be supposed that the SA**1**–**4** represent promising candidates for the novel SIRT1 activators. By employing an integrative network pharmacology that combines CTD and PPI approaches, a connection between anthranilate sulfonamides and the possible signaling pathways was extensively extended through the in silico and in vitro studies. GO and KEGG analyses showed that the targets of our derivatives involve the regulation of ADAM10 in the non‐amyloidogenic cascade (Khezri et al. 2023) and CDK5 in tau phosphorylation (Kimura et al. 2014), which could reduce the generation of both AD hallmarks (i.e., amyloid plaques and neurofibrillary tangles in the brain). Additionally, the downstream pathway of SIRTs was also revealed through the involvement of CAPN1 (Mahaman et al. 2019), PI3K (Gabbouj et al. 2019), NFKB, iNOS, and COX2 (Bhattacharya et al. 2023), in modulating calcium homeostasis, insulin‐mediated glucose uptake, and AGEs‐RAGE axis. Over‐calcium elevation has been documented as one of the complications that evokes free radical production and alters mitochondrial oxidative stress in neuronal cells, leading to the disruption of calcium homeostasis in AD. This further highlighted their comprehensive capabilities, contributing to the anti‐AD neuropathology as well as the crosstalk of diabetes mellitus and cardiovascular diseases (Chakraborty et al. 2021).

**FIGURE 10:**
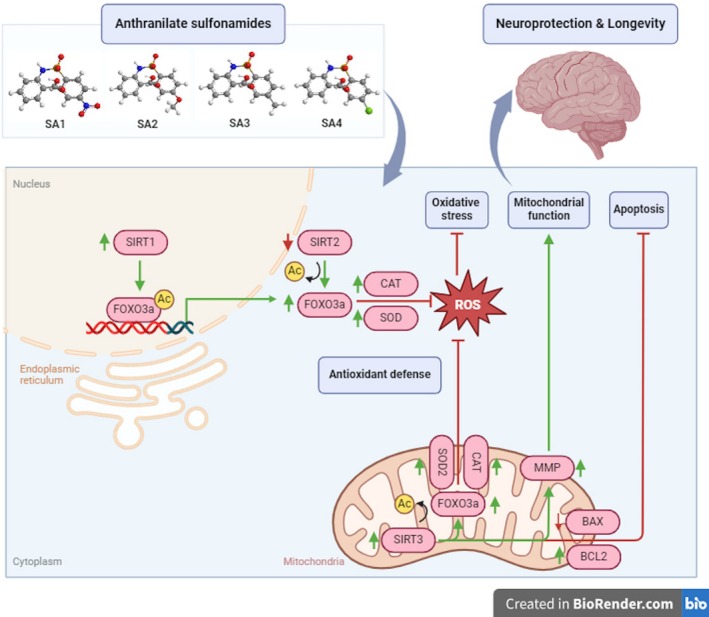
Schematic representation of the neuroprotective effects of anthranilate sulfonamides (SA**1–4**) on hydrogen peroxide (H_2_O_2_)‐mediated oxidative damage in human neuronal SH‐SY5Y cells created in https://www.biorender.com. Ac is denoted as acetylation.

Taken together, the revealed neuroprotective mechanisms of these four AA‐linked sulfonamides (SA**1**–**4**) suggested their superior potentials to the parent AA. Our findings are in concordance with the recent clinical trials, which demonstrate the therapeutic potentials of AA derivatives and their related tryptophan metabolites on improving cognitive deficit in many neurological patients, including bipolar disorder (Birner et al. 2017), depression (Ryan et al. 2020), stroke (Bakker et al. 2023), and AD (Cespedes et al. 2022). Interestingly, the investigated compounds (SA**1**–**4**) displayed multiple actions on restoring cellular antioxidant capacity, preventing apoptosis, and maintaining MMP through SIRT1/2/3‐FOXO3a signaling cascades (Figure 10). This suggested that AA‐linked sulfonamides could serve as pharmacophores for further rational design and structural modification, which may extend a high degree of molecular diversity to enhance structural variations and pharmacological profiles against neurodegeneration, especially combating and delaying the pace of AD.

## 5 Conclusion

A set of four synthesized AA‐linked sulfonamides (SA**1**–**4**) was investigated for their neuroprotective effects. The compounds displayed their abilities to maintain cellular antioxidant capacity, mitochondrial function, and anti‐apoptotic event against oxidative injury in human neuronal SH‐SY5Y cells. The compounds protected the injured cells by maintaining SIRT1 deacetylated activity and modulating SIRT1/2/3‐FOXO3a signaling cascades. Supported by the molecular docking study, the compounds could act as SIRT1 modulators, exhibiting similar binding modes to those of the RSV, and mainly governed by pi‐interactions and hydrogen bonding via ILE223, ASN226, and ARG446. Notably, the unique structural features of these sulfonamide‐containing compounds (i.e., terminal benzene ring, sulfonyl oxygen, and carboxylic group of AA) could contribute to preferable binding affinity towards the SIRT1 target. Additionally, the predictions suggested that all compounds could be further developed as oral neuroprotective drugs with good pharmacokinetics and relatively safe profiles. Combined with the perspective of network pharmacology, these SA**1**–**4** SIRT1 activators with multifunctional effects on regulating several downstream pathways via SIRTs‐FOXO3A cascades might be an alternative for AD remedies and extension of life expectancy. However, further efficient advanced in vivo and clinical studies are recommended for successful development.

## Author Contributions

**Waralee Ruankham:** investigation, methodology, formal analysis, writing – original draft, writing – review and editing. **Veda Prachayasittikul:** methodology, investigation, formal analysis, writing – review and editing. **Ratchanok Pingaew:** methodology, writing – review and editing, formal analysis, investigation, resources. **Wilasinee Jeungprasopsuk:** methodology, investigation, formal analysis, writing – review and editing. **Tanawut Tantimongcolwat:** methodology, investigation, formal analysis, writing – review and editing, funding acquisition. **Virapong Prachayasittikul:** writing – review and editing, funding acquisition. **Supaluk Prachayasittikul:** conceptualization, writing – review and editing, funding acquisition, resources. **Kamonrat Phopin:** conceptualization, methodology, investigation, formal analysis, supervision, resources, writing – review and editing, funding acquisition.

## Funding

This study was supported by Mahidol University (Fundamental Fund: fiscal year 2025 by the National Science Research and Innovation Fund (NSRF)),Thailand.

## Conflicts of Interest

The authors declare no conflicts of interest.

## Supporting information


**Appendix S1:** jnc70377‐sup‐0001‐AppendixS1.pdf.

## Data Availability

The data that support the findings of this study are available from the corresponding author upon reasonable request.
