# Supplementary material for: Unraveling Network Pharmacology‐Based Therapeutics of Anthranilate Sulfonamides via Sirtuins/FOXO3a Cascade in Alzheimer's Disease
Source: J Neurochem. 2026 Feb 19;170(2):e70377. doi: 10.1111/jnc.70377 (PMC12920268; doi:10.1111/jnc.70377)
Supplement: Supplementary file 1 — Appendix S1: jnc70377‐sup‐0001‐AppendixS1.pdf. [file JNC-170-0-s001.pdf]

Supplementary Data

Unraveling Network Pharmacology-based Therapeutics of Anthranilate Sulfonamides *via* Sirtuins/FOXO3a Cascade in Alzheimer's Disease

Waralee Ruankham[1], Veda Prachayasittikul[2], Ratchanok Pingaew[3], Wilasinee Jeungprasopsuk[2], Tanawut Tantimongcolwat[2], Virapong Prachayasittikul[4], Supaluk Prachayasittikul[2], Kamonrat Phopin[2,4,*]

[1]Department of Clinical Chemistry, Faculty of Medical Technology, Mahidol University, Bangkok 10700, Thailand

[2]Center for Research Innovation and Biomedical Informatics, Faculty of Medical Technology, Mahidol University, Bangkok 10700, Thailand

[3]Department of Chemistry, Faculty of Science, Srinakharinwirot University, Bangkok 10110, Thailand

[4]Department of Clinical Microbiology and Applied Technology, Faculty of Medical Technology, Mahidol University, Bangkok 10700, Thailand

*Corresponding author: Kamonrat Phopin

E-mail: kamonrat.php@mahidol.ac.th

Phone: +66 (2) 441 4376, Fax: +66 (2) 441 4380

Table S1. Comparative docking results of anthranilate sulfonamides.

Ligand	Binding free energy (kcal/mol)	Interacting type	Bonding interaction	Interacting amino acids	Bond distance (Å)
SA1	–8.53	Hydrogen bonding	Conventional	HIS2	2.0768
				LYS3	2.3558
				ASN226	2.9511
			Carbon	ARG446	3.1266
		Hydrophobic bonding	Pi-Pi Stacked	FDL4	4.5695
			Pi-Alkyl	ILE223	4.6548
				PRO447	4.9877
				LEU450	5.4153
SA2	–8.26	Hydrogen bonding	Conventional	HIS2	2.0689
				LYS3	1.9695
				ASN226	2.9373
			Pi-Donor	ASN226	2.8614
			Carbon	PRO447	3.6601
		Hydrophobic bonding	Pi-Alkyl	FDL4	4.6432
				ILE223	4.9102
				ARG446	4.4829
SA3	–8.54	Hydrogen bonding	Conventional	HIS2	1.9588
				LYS3	1.9488
				ASN226	2.7267
			Pi-Donor	ASN226	2.9311
		Hydrophobic bonding	Alkyl	LEU215	5.2125
			Pi-Alkyl	FDL4	4.5269
				ILE223	4.9143
				ARG446	4.2789
SA4	–8.38	Hydrogen bonding	Conventional	HIS2	1.8537
				LYS3	1.7765
				ASN226	2.7538
			Pi-Donor	ASN226	2.6634
		Hydrophobic bonding	Pi-Alkyl	FDL4	5.0310
				ILE223	5.0243

				ARG446	4.5569
AA	–4.86	Hydrogen bonding	Conventional	LYS3	1.6127
				GLU300	2.0592
				GLY415	1.7867
		Hydrophobic bonding	Pi-Alkyl	LEU215	5.3654
RSV	–7.57	Hydrogen bonding	Conventional	LYS3	1.8052
				GLU230	1.9490
			Pi-Donor	ASN226	3.1598
		Hydrophobic bonding	Pi-Pi Stacked	FDL4	3.7805
			Pi-Sigma	ILE223	3.8871
			Pi-Alkyl	LEU202	5.0386
				ARG446	3.9994

Table S2. Centrality analyses of the top 20 targets of anthranilate sulfonamides.

Protein	Gene	Pathway	Degree	Betweenness	Closeness
Signal transducer and activator of transcription 3	STAT3	Cellular responses to interleukins, KITLG/SCF, LEP, and growth factors.	17	75.09341	0.207865
Hypoxia inducible factor 1 subunit alpha	HIF1A	Hypoxia, embryonic vascularization, tumor angiogenesis, and ischemic disease.	16	263.5324	0.211429
Nuclear factor kappa B subunit 1	NFKB1	Inflammation, immunity, differentiation, cell growth, tumorigenesis, and apoptosis.	15	155.8746	0.20442
NAD-dependent protein deacetylase sirtuin 1	SIRT1	Cell cycle, response to DNA damage, metabolism, apoptosis, and autophagy.	14	95.9483	0.203297
Prostaglandin-endoperoxide synthase 2	PTGS2	Prostanoid synthesis, increased cell adhesion, phenotypic changes, resistance to apoptosis, and tumor angiogenesis.	13	85.11738	0.203297
Platelet-derived growth factor receptor beta	PDGFRB	Embryonic development, cell proliferation, survival, differentiation, chemotaxis, and migration.	12	124.5995	0.197861
Signal transducer and activator of transcription 1	STAT1	Cellular responses to interferons (IFNs), cytokine KITLG/SCF, and growth factors.	12	76.54984	0.197861

Serpin family E member 1	SERPINE1	Fibrinolysis down-regulation, cell adhesion, and cell migration.	11	128.4542	0.202186
Matrix metallopeptidase 3	MMP3	Degradation of fibronectin, laminin, gelatins, and collagens, and cartilage proteoglycans.	11	15.79522	0.2
Nitric oxide synthase 2	NOS2	Mediation of tumoricidal and bactericidal actions, and inflammatory stimulus-dependent S-nitrosylation.	9	6.800433	0.193717
Phosphoinositide-3-kinase regulatory subunit 1	PIK3R1	Glucose uptake, glycogen synthesis, and nuclear translocation.	9	12.63556	0.188776
Protein tyrosine phosphatase non-receptor type 11	PTPN11	Signal transduction from the cell surface to the nucleus.	8	30.78115	0.194737
Nuclear factor erythroid 2-related factor 2	NFE2L2	Oxidative stress	8	2.108438	0.19171
Solute carrier family 2 member 1	SLC2A1	Facilitative glucose transporter into the brain.	8	83.62338	0.194737
Telomerase reverse transcriptase	TERT	Replication of chromosome termini.	7	5.261539	0.190722
Matrix metallopeptidase 1	MMP1	Cleavage of collagens.	6	0	0.189744

Disintegrin and metalloproteinase domain-containing protein 10	ADAM10	Proteolytic release of several other cell-surface proteins, including heparin-binding epidermal growth-like factor, and regulated alpha-secretase cleavage of amyloid precursor protein (APP).	6	27.23813	0.189744
Cyclin-dependent kinase 5	CDK5	Neuronal cell cycle arrest, differentiation, and apoptotic cell death	5	54.89214	0.192708
Cathepsin D	CTSD	APP processing following cleavage and activation by ADAM30, leading to APP degradation.	4	134.2481	0.186869
Calpain 1	CAPN1	Cytoskeletal remodeling and signal transduction.	4	246.4462	0.192708

Table S3. Physicochemical parameters of anthranilate sulfonamides.

**Properties**	**SA1**	**SA2**	**SA3**	**SA4**	**AA**
Formula	$C_{13}H_{10}N_2O_6S$	$C_{14}H_{13}NO_5S$	$C_{14}H_{13}NO_4S$	$C_{13}H_{10}ClNO_4S$	$C_7H_7NO_2$
Molecular Weight	322.29	307.32	291.32	311.74	137.14
Rotatable Bonds	5	5	4	4	1
H-bond Acceptors	6	5	4	4	2
H-bond Donors	2	2	2	2	2
Polar Surface Area	137.67	101.08	91.85	91.85	63.32
Lipophilicity	2.32	2.32	2.89	2.76	1.21
Molar Refractivity	79.34	77.01	75.48	75.52	37.81
Lipinski	Yes	Yes	Yes	Yes	Yes
Veber	Yes	Yes	Yes	Yes	Yes
Ghose	Yes	Yes	Yes	Yes	No

Table S4. Predicted ADMET parameters of anthranilate sulfonamides.

Properties	SA1	SA2	SA3	SA4	AA
**Absorption**					
Water Solubility	Moderate	Moderate	Moderate	Moderate	High
Caco-2 Permeability	Moderate	High	High	High	Moderate
Intestinal Absorption	High	High	High	High	High
Skin Permeability	High	High	High	High	High
**Distribution**					
Human VDss	Low	Low	Low	Low	Low
BBB Permeability	Yes	Yes	Yes	Yes	Yes
Fraction Unbound	0.16	0.221	0.229	0.219	0.517
CNS Permeability	Moderate	Moderate	Moderate	Moderate	Moderate
**Metabolism**					
CYP2D6 Substrate	No	No	No	No	No
CYP3A4 Substrate	No	No	No	No	No
CYP1A2 Inhibitor	No	No	No	No	No
CYP2C19 Inhibitor	No	No	No	No	No
CYP2C9 Inhibitor	No	No	No	No	No
CYP2D6 Inhibitor	No	No	No	No	No
CYP3A4 Inhibitor	No	No	No	No	No
**Excretion**					
Total Clearance	0.424	0.545	0.571	0.021	0.587
Renal OCT2 Substrate	No	No	No	No	No
**Toxicity**					
AMES Toxicity	No	No	No	No	No
Max. Tolerated Dose	High	High	High	High	High
Oral Rat Acute Toxicity	1.886	2.139	2.027	2.039	1.543
Hepatotoxicity	Yes	No	No	No	No
Skin Sensitization	No	No	No	No	No

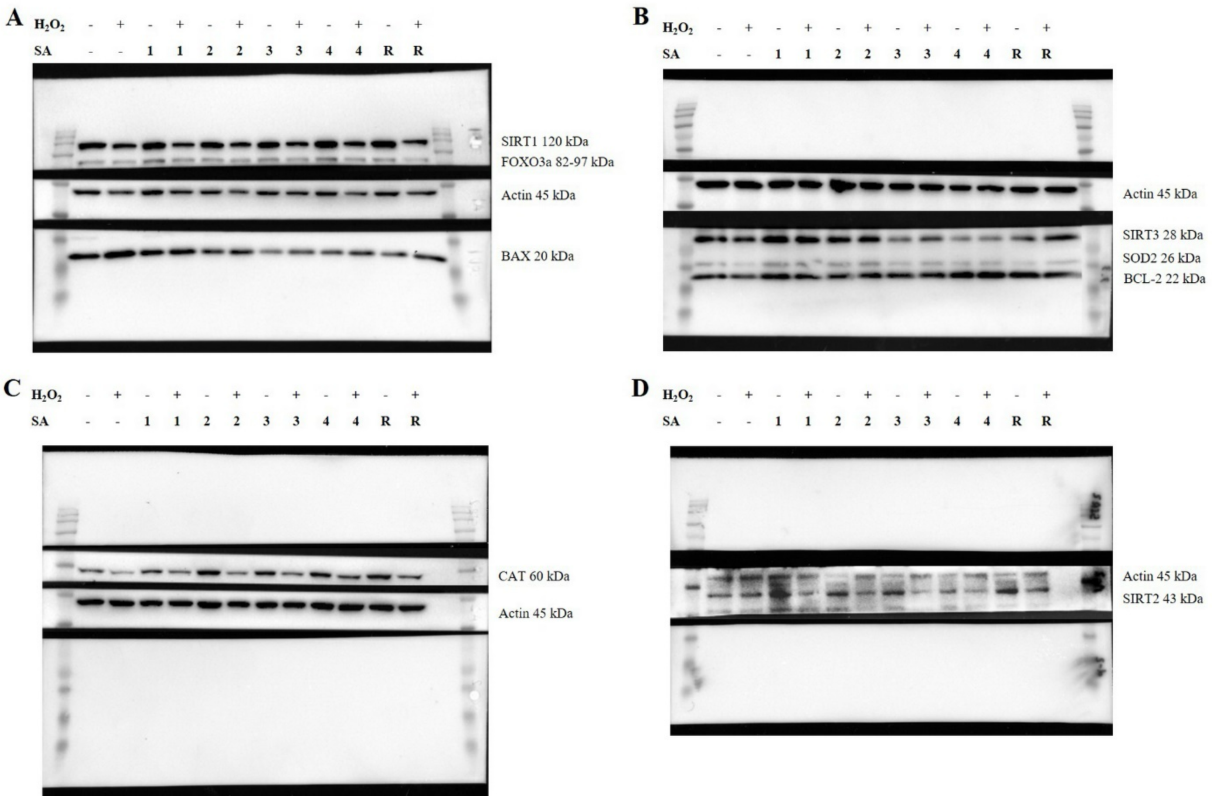

Figure S1. Original western blot with merged protein marker and protein of interest. Each part of the blot separately presented the band intensity of a specific protein at different time exposures.
